# Microfluidic lab-on-chip design for efficient relative humidity sensing using a capacitive transducer

**DOI:** 10.1038/s41598-025-10701-y

**Published:** 2025-07-17

**Authors:** Mohamed Abdelghani, Osama Hussien

**Affiliations:** 1 Faculty of EMS, German University in Cairo, Cairo, Egypt; 2 Faculty of IET, German University in Cairo, Cairo, Egypt

**Keywords:** Biomedical applications, Capacitive transducer, Humidity, Hygrometer, Lab on chip, Relative permittivity, Biomedical engineering, Electrical and electronic engineering

## Abstract

Recent advancements in microelectronics have provided substantial motivation for ongoing innovation within the domain of sensor and measurement technologies. The application of humidity sensors across diverse settings has been integral to system monitoring initiatives. There exists potential for further enhancements aimed at the development of sensors that are not only more efficient and safer but also more conducive to human interaction. The sensors thus developed and presented in this article hold promise for deployment in pioneering applications, offering precision measurements surpassing those of traditional sensors. Moreover, these advanced sensors are poised to be integral components of emergent applications in biomedical research and energy harvesting technologies. In this study, a lab-on-a-chip (LOC) design that uses microfluidic principles is designed and implemented. The proposed design is concerned with measuring the relative humidity of the surrounding medium using a capacitive transducer. The proposed methodology is designed, implemented, and tested on a low-cost experimental setup; results were recorded using LabVIEW. Achieving a resolution of 33.993 mL/V with a sensitivity of 0.0596 V m/F and noise tolerance of 2.028 V, the system successfully demonstrated its capability by implementing a prototype that charges a 22 pF capacitor. While the observed capacitance change and corresponding voltage output remain too low for direct device charging, this proof-of-concept demonstrates the potential of harvesting ambient moisture-driven energy. Further work on materials and circuit design will be needed to quantify power output and advance toward sustainable mobile-device charging.

## Introduction

A lab-on-a-chip is basically a very small device that can perform laboratory functions on a very small scale, often at the size of a single chip. They are designed to be portable, cost-effective and capable of performing complex analyses with very small sample sizes. Essentially, they miniaturize and streamline traditional laboratory procedures onto a small, portable chip. Humidity is a physical parameter that describes the amount of water vapor present in a gas that can be a mixture, such as air, or a pure gas, such as nitrogen or argon. Humidity can be measured in both an absolute and relative manner. Consequently, it is critically important in various fields of scientific and industrial applications, including hospitals, laboratories, computer warehouses, and food processing^1^.

Humidity sensors are divided into two types: Relative humidity (*RH*) sensors and absolute humidity (moisture) sensors. Most humidity sensors are relative humidity sensors, which can be further classified into ceramic, semiconductor, and polymer humidity sensors^2^. Available absolute humidity sensors (hygrometers) are solid moisture sensors and mirror chilled hygrometers. Humidity, in general, can be measured using absolute humidity, dew point, mixing ratio, and relative humidity. Among these measurements, relative humidity is the most common measure used in the literature^1,3–6^. In a closed system, *RH* can be defined as the ratio of the partial pressure of water vapor present in the air to the saturation vapor pressure of water at a given temperature. It is generally expressed by the formula

1 $$\begin{aligned}{RH} = \frac{{P}_{t}}{{P}_{s}}, \end{aligned}$$

where *P*_*t*_ is the partial pressure and *P*_*s*_ is the saturated vapor pressure^5^. *RH* is a function of temperature, and thus it is a relative measurement. The *RH* measurement is expressed as a percentage.

### Problem statement

The variable capacitance value is dependent on four important parameters. They are the distance between the plates of the variable capacitor, occupying area of the plates, the permittivity of the free space, the relative permittivity, and the dielectric material. These parameters can be used to vary the capacitance value of the variable capacitor. Various research^1,7^ was oriented towards controlling these parameters but was not efficient in harvesting the energy of water vapor in a dielectric medium. This is done by controlling the permeability of the dielectric medium of a capacitor transducer for humidity.

### Contributions

The primary contributions of this work include:

- Development of a novel capacitive humidity sensor with enhanced sensitivity.
- Implementation of a LOC approach for energy harvesting from water vapor.
- Demonstration of a proof-of-concept for capacitor charging using environmental moisture.

### Organization

In Section “Related works”, the various approaches to using LOC implementations have been presented. In section “Experimental work”, the methodology is explained, and both the experimental data and experimental setup are shown. In section “Results”, the results are presented and discussed. In section “Conclusions”, the conclusion of this study is made, and future recommendations for modifications and developments are introduced.

## Related works

Humidity sensing has seen remarkable progress in recent years, with scientists experimenting with new materials and designs to create better tools for measuring environmental moisture. From small sensors in microchips to flexible bio-materials, researchers are constantly refining how these devices detect and analyze humidity.

Table 1 presents the research works that developed more complex humidity sensors by controlling the characteristics of various sensors (such as piezoelectric humidity sensing and electrical tunneling through temperature-stabilized nanometer gaps)^1,8^. Today, humidity sensors are made of a variety of materials, including electrolytes, organic polymers, and ceramic pads^4^. In^1^, the authors reviewed three types of Micro-Electro-Mechanical System (MEMS) humidity sensors: capacitive, Piezoelectric, and resistive sensors. While capacitive sensing depends on the changing permittivity of the sensing material, the humidity can be determined in the piezoelectric sensors by measuring the shift in the resonance frequency. The resistive sensors use the change in resistivity to detect the humidity change.

**Table 1:** Comprehensive review of capacitive sensor research approaches.

References	Approach	Key characteristics	Limitations
Proposed design	LOC design for a capacitive relative humidity sensor based on a capacitive transducer implementation	Change in capacitance with respect to change in relative humidity in the surrounding	Residual moisture and manual vapor control led to variability and potential measurement inaccuracies
Alfaifi et al.^1^	MEMS humidity sensors	Multiple types: capacitive, piezoelectric, resistive	Gas contamination sensitivity
Zhang et al.^6^	Printed flexible capacitive sensors	Field-deployable form factor	Potential manufacturing challenges
Luka et al.^7^	Microfluidics-LOC biosensors	Enhanced system efficiency, integrated chip design	Integration complexity
Banerjee et al.^8^	Temperature-compensated tunneling humidity sensor	Broad output range, minimal temperature sensitivity	Temperature fluctuation challenges
Al-Ta’ii et al.^9^	DNA-based sensors	Flexible bio-material, high sensitivity and accuracy	Limited to specific environmental conditions
Dokmeci & Najafi^10^	Capacitive sensor for micro-packages	High-sensitivity, precise moisture detection	Restricted to specific packaging environments
Hirama & Komazaki^11^	Microfluidic humidity-driven energy harvester with multi- element integration	10$\times$ voltage boost via integrated cation-exchange membranes	Requires photolithography, specialized membranes, and clean-room facilities
Li et al.^12^	Computational optimisation of interdigital capacitive sensor geometry	Models predict optimal 3 $\upmu$m electrodes with 14 $\upmu$m spacing	No physical devices; practical realisation needs e-beam lithography or high-resolution masks
Zhou et al.^13^	CMOS-compatible Al–Mo bilayer capacitive sensor fabrication	High sensitivity (0.4264 pF/% RH) and 850 ms response from multi-step CMOS flow	Complex process: Si oxidation, metal evaporation, polyimide spin-coat, photolithography available only in semiconductor fabs
Hu et al.^14^	Microfluidic capacitive sensor modelling for RBC detection	25 $\upmu$m PDMS channels optimised for electric-field distribution	Precise lithography for channel definition; fabrication complexity remains a barrier

While earlier work focused on making sensors more accurate and durable, there is still unexplored potential in using humidity itself, specifically, the way moisture changes the air’s electrical properties as a source of energy^1,6–10^. This research is founded on filling this gap, aiming to develop methods that not only detect humidity but also re-purpose its dielectric dynamics as a sustainable energy resource.

Capacitive sensors in general exhibit higher linearity, faster response, and temperature compensation but are sensitive to gas contamination compared to resistive sensors^1^. Piezoelectric sensors, on the other hand, do not require an external power source which is needed for both capacitive and resistive. The resistive sensors are cheaper to build and have a simple readout circuit compared to the other two types.

In^8^, the authors presented the design, fabrication, electrical characterization, and working of a temperature-compensated tunneling humidity sensor. Fabrication and response of a humidity sensor based on electrical tunneling through temperature-stabilized nanometer gaps. The article in^8^ introduces a novel micro-engineered humidity sensor capable of offering a broad output range with minimal sensitivity to temperature fluctuations. The sensor developed in^8^ employs a polymer’s expansion upon exposure to humidity, akin to capacitive devices, yet generates a resistive output to gauge the polymer’s expansion via tunneling current across a humidity-responsive, thermally steady nanogap.

In^9^, Deoxyribonucleic acid (DNA)-based sensors, especially humidity and alpha particle sensors, have become quite popular in recent times due to the flexible and highly optimizable nature of this fundamental bio-material. The application of DNA electronics allows for more sensitive, accurate, and effective sensors to be developed and fabricated.

In^10^, the authors present the fabrication and characterization of a high-sensitivity polyimide-based humidity sensor for monitoring internal humidity levels in anodic-bonded hermetic micro-packages. This capacitive sensor utilizes CU1512 polyimide film to sense moisture with remarkable precision. However, the work in^10^ distinguishes itself by implementing an LOC approach specifically targeting energy harvesting from water vapor. While previous researchers focused primarily on sensing and measurement, this article explores the potential of converting humidity into harvestable energy, opening up new possibilities for environmental and portable power solutions.

With the continuous development of sensing technology, different innovations have been made to increase the efficiencies of proposed sensor designs and fabrications. Innovations in controlling the relative permeability of capacitive transducers not only have provided the chance to engage modern technologies of LOCs and microfluidics but also opened the road for merging them to fabricate and design more efficient, user and environment-friendly sensors.

Microfluidics is the science of precise control and manipulation of fluids that are geometrically constrained to a small scale. Therefore, the concept of Microfluidics is widely used in different applications ranging from micro-scaled medical tests to breakthrough innovative research projects. Such projects include Pharmaceutical Engineering, Biotechnology, Patient Testing, and Diagnostics, Bio-defense, and Chemical Engineering. The innovation of microfluidics also has paved the path for the possible integration with LOC technologies.

Such integration has highly enhanced the efficiency of different system designs that include fluids and can be all integrated on a chip.

The authors in^7^ reviewed biosensors that are based on integrating the microfluidics with LOCs. In this study, a novel design of a *RH* variable capacitive transducer is proposed. The design proposes a LOC technology combining microfluidics to measure *RH* in closed environments.

Finally, In^4^, researchers described the fabrication and performance of a polyimide-based humidity sensor tailored for monitoring internal humidity in hermetically sealed micro-packages. These three researchers built up the proposal of harvesting electricity from a humidity-based sensor sensitive to environmental changes in humidity in a fabricated LOC design.

Table 1, summarizes the various fabricated capacitive transducers along with their respective implementation methods, tailored to the specific measurement approach or intended application.

Recent advances in humidity sensing and energy harvesting demonstrate diverse approaches with varying complexity-performance trade-offs. Most notably, Hirama and Komazaki^11^ developed a microfluidic humidity-driven energy harvester remarkably similar to our concept, achieving a 10-fold voltage improvement through multi-element integration. However, their approach requires photolithography equipment and specialized cation-exchange membranes, materials typically used in fuel cells and water treatment systems that require careful handling and specialized suppliers. While their sophisticated design achieves superior performance, it necessitates cleanroom access and advanced fabrication capabilities.

Li et al.^12^ provided valuable theoretical insights through extensive modeling, demonstrating that optimal performance requires 3 $\mu$m electrode features with 14 $\mu$m spacing. However, their work remained entirely computational, no physical sensors were fabricated. Translating their theoretical 3 $\mu$m features into reality would require electron-beam lithography or high-resolution photomasks, further emphasizing the gap between theoretical optimization and practical implementation.

Zhou et al.^13^ successfully fabricated high-performance sensors through a complex CMOS-compatible process involving silicon oxidation, metal evaporation (Mo/Al bilayers), polyimide synthesis and spin-coating, precise thermal ramping (150 $^{\circ }$C$\rightarrow$250 $^{\circ }$C$\rightarrow$300 $^{\circ }$C, over 115 min), and photolithographic patterning. While achieving excellent performance (0.4264 pF/%RH, 850 ms response), their process requires multiple specialized equipment pieces including oxidation furnaces, metal evaporators, spin coaters, and photolithography systems, infrastructure typically found only in semiconductor fabrication facilities.

In the broader context of microfluidic sensors, Hu et al.^14^ demonstrated that even in different applications (RBC detection), the fabrication complexity remains a barrier. Their 25 $\mu$m PDMS microfluidic channels required precise lithographic techniques to achieve optimal electric field distributions for bioparticle detection.

Our approach deliberately prioritizes accessibility over peak performance. By using laser-cut acrylic (2 mm features vs. 2–3 $\mu$m in cited works) and commercial aluminum sheets, we achieve functional humidity-driven energy harvesting using equipment available in most university makerspaces and small laboratories. While our 275 pJ energy storage and 0.0596 V$\cdot$m/F sensitivity cannot match the sophisticated devices above, our method enables rapid prototyping without cleanroom access. This trade-off is intentional, we demonstrate that humidity energy harvesting, a concept proven effective by Hirama and Komazaki, can be made accessible to researchers and students worldwide without specialized semiconductor processing facilities.

The significance lies not in competing with photolithographic precision, but in democratizing access to this emerging technology. Where Hirama and Komazaki’s breakthrough design requires specialized facilities and materials with limited suppliers, our approach enables same-day fabrication and iterative design improvements, making it particularly valuable for educational purposes and resource-constrained research environments.

## Experimental work

This section outlines the experimental work conducted to evaluate the performance of the capacitive sensor developed in this work. To begin, the experimental work is introduced to obtain the calibration curve, which is essential for determining the sensitivity and resolution of the sensor. This process requires the execution of a total of ten experiments, each designed to provide critical intuition into the sensor’s behavior under varying conditions.

The first five experiments involve boiling water in a conical flask on an electric heater at 250 $^{\circ }$C at different starting volumes. Once an observable volumetric change is observed, time is recorded (x minutes). For every experiment, five volumetric readings will be recorded every x minutes. The recorded values are then inserted into a spreadsheet to obtain volume versus time plots.

The equation of the best-fit line of each plot is then obtained and an average gradient of all five volume versus time plots is calculated. This can be calculated by adding the gradient of each plot and dividing by the number of experiments (which is five in the proposed methodology).

**Fig. 1:**
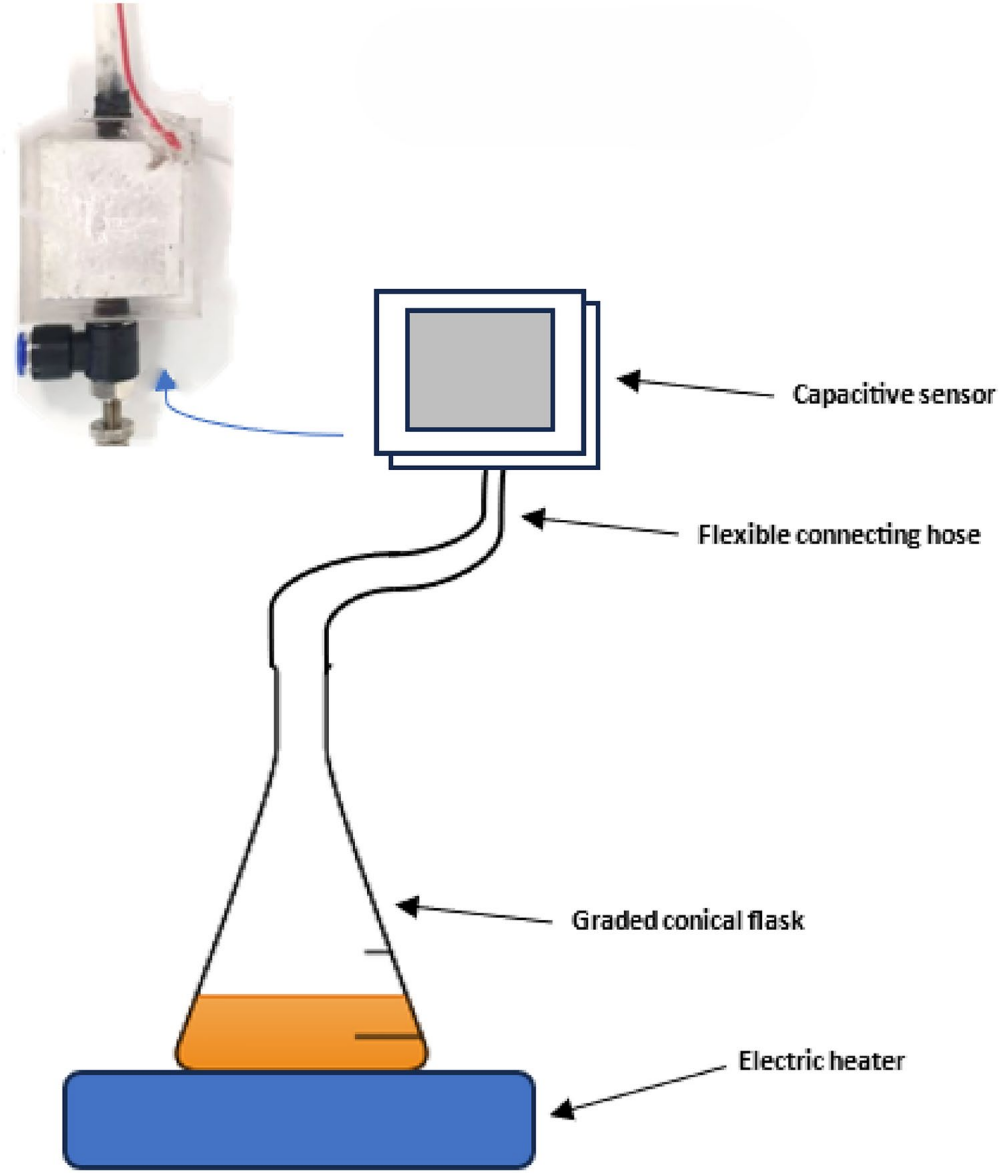
Collection of water vapor through a volumetric setup.

The final five experiments involve carrying out the same procedure, however, in such a case, the capacitive sensor will be connected to the conical flask as in Fig. 1 and the first five experiments will be repeated with the different starting volumes at different time intervals recorded for each experiment (x minutes).

The voltage output curve is then obtained in real time using the LabVIEW ®set-up. LabVIEW ®works as follows. A signal generator with 14 V and 50 kHz frequency is connected to the capacitive transducer circuit. The output of this circuit is connected to the voltage multiplier circuit, which takes as an input the output of the capacitive transducer circuit and passes it to the rectifier. The rectifier then converts it to a DC voltage and then passes it on to the multiplier to increase the voltage. The output of the voltage multiplier circuit is then connected to an Arduino ®Uno ®, and then the inputted voltage is passed into LabVIEW ®to sketch voltage versus time plots. This setup can be seen in Fig. 7. The curves obtained are exported into a spreadsheet and the equation of each curve can then be obtained to assess the sensitivity and thus, the resolution of the sensor.

To plot volume versus time, a conical flask filled with a certain volume of water is connected to the capacitive sensor via a delivery tube as visible in Fig. 1. Since small graded conical flasks were unavailable. An ungraded conical flask was graded by filling water of a known volume using a syringe and marking the known volume added using a marker. The conical flask shape was exactly chosen since it has a stable base, and the delivery tube can be fitted to it.

### Sensor fabrication

**Fig. 2:**
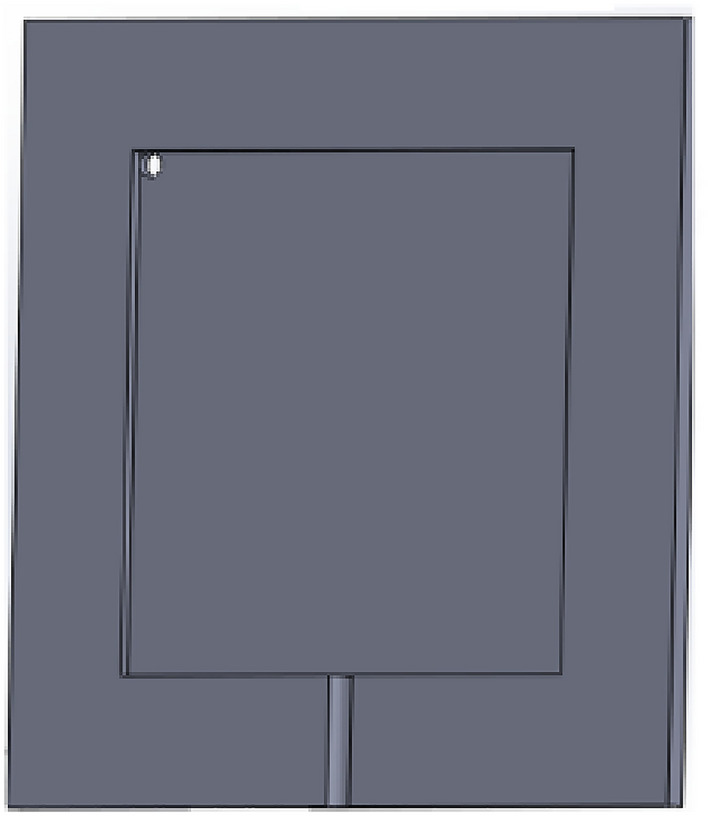
Isometric view of the fabricated sensor.

The capacitor transducer is in the shape of a cuboid with an internal cuboid gap that separates the two aluminum sheets connected with jumper wires. The design of capacitive transducer was designed on SolidWorks ®and divided into several parts. Then the parts are exported as a Dxf file. The file is then imported into a Laser cutting machine and cut on an acrylic sheet for a fine surface finish. An isometric view of the final design of the fabricated capacitive humidity can be seen in Fig. 2.

**Fig. 3:**
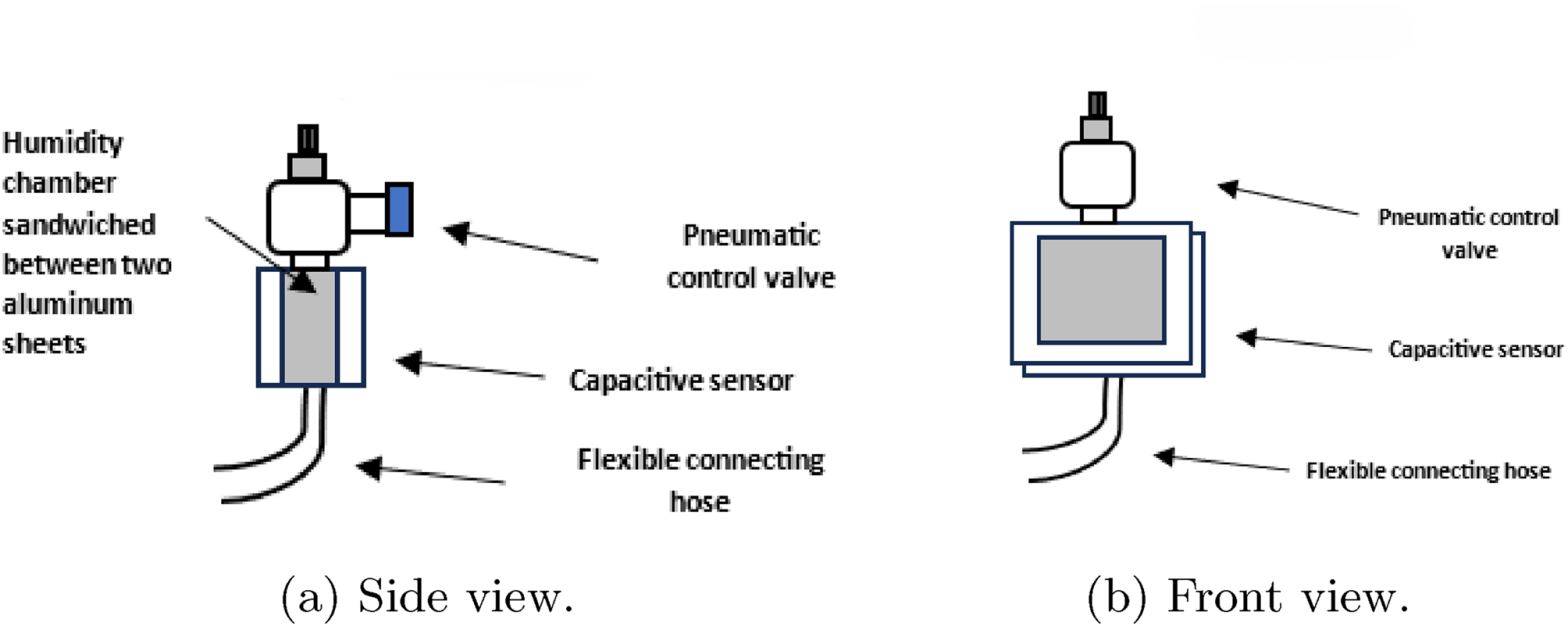
Side and front views of the fabricated capacitive humidity sensor.

As visible in Fig. 3a and b, the cuboid has one inlet port and one external port. The inlet port is for the dielectric vapor to enter the capacitive transducer through the delivery tube. However, the outlet port is connected to a flow control valve. The flow control valve gives us the ability to control the percentage of the dielectric vapor leaving the transducer.

Subsequent sections of this document will express that the capacitive sensor currently under design exhibits markedly low sensitivity, which makes it incapable of detecting extremely diminutive capacitance values. As described by the governing equation of capacitance, which has been explained heretofore, increasing the capacitance necessitates a reduction in the interstitial distance between the dual conductive plates, coupled with an amplification of their respective surface areas.

Before the fabrication of the capacitive humidity sensor with revised dimensions, an array of dimensions was empirically evaluated using a fundamental capacitive transducer, employing paper as a dielectric medium. This preliminary investigation aimed to determine the optimal dimensions that would yield the highest sensitivity through a methodical process of trial and error. The peak of these trials indicated that a marginally larger area than that of the initial design, paired with a reduced gap between the conductive plates from 10 mm to 2 mm, was most effective.

**Fig. 4:**
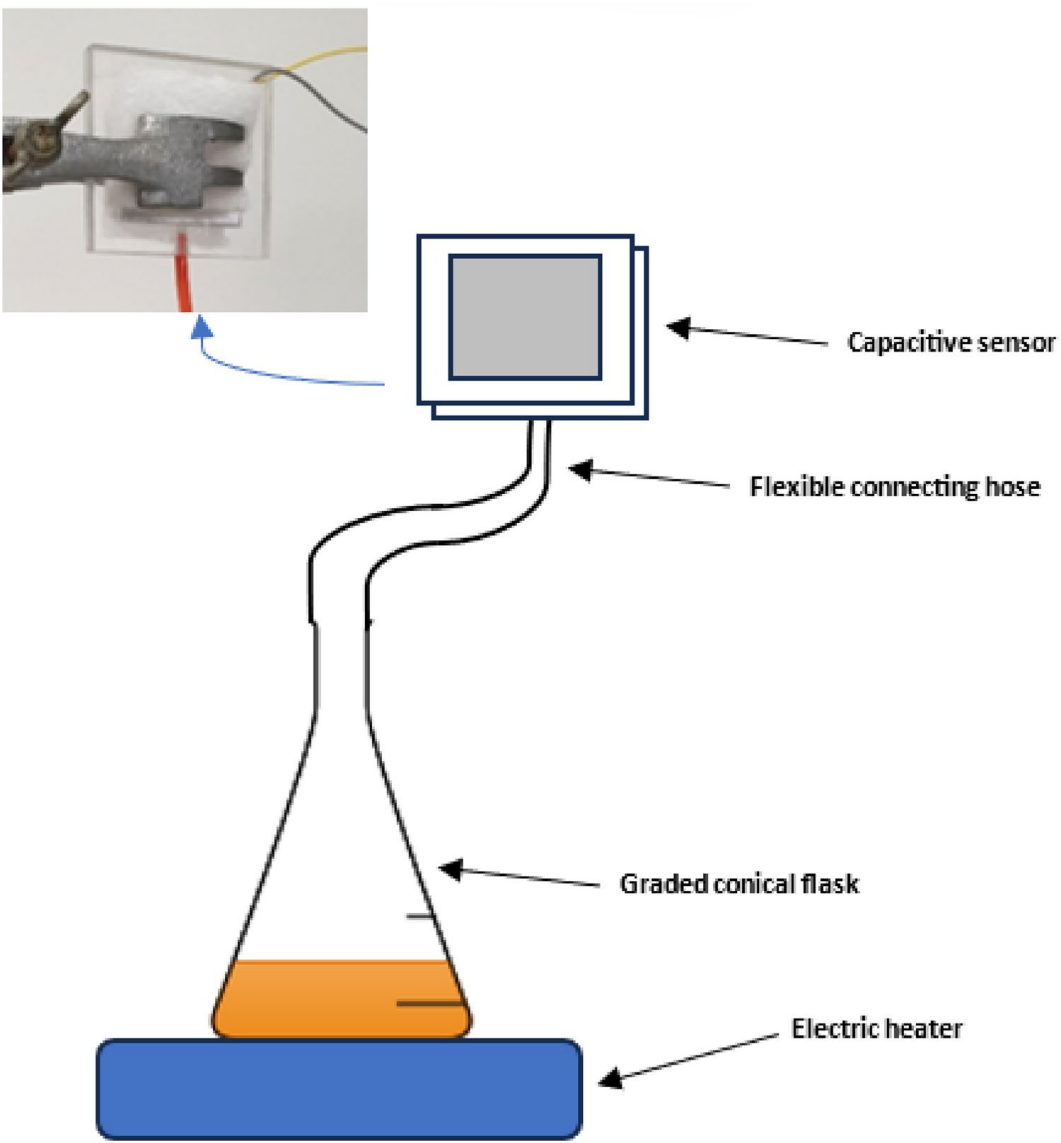
Volumetric setup of the capacitive sensor with the new dimensions.

Accounting for the thickness of the aluminum sheets (two sheets, each 0.016 mm) and the double-sided tape (two layers, each 0.2 mm) utilized to secure the aluminum sheets within the capacitor sensor, the resultant distance is precisely 1.568 mm. Figure 4 illustrates that the diameter of the delivery tube was excessively large for insertion into the capacitive sensor. Consequently, a linkage system was designed to facilitate the conveyance of water vapor from the larger aperture to a significantly smaller one. This system comprises an 8 mm flexible hose affixed to the delivery tube, which is in turn connected to a 3D-printed intermediary designed to bridge the 8 mm flexible hose and a 4 mm pneumatic hose.

In this design iteration, the outlet port was omitted to maximize the volume of water vapor introduced under pressure into the capacitive sensor. The inlet port was intentionally not sealed with epoxy to allow for the evaporation of residual water from the previous experiments by exposing the sensor to sunlight. This ensures that each new experiment begins with a thoroughly dry capacitor. Any remaining moisture from prior experiments could erroneously elevate the initial capacitive readings, thereby compromising the results. The design was conceptualized in SolidWorks ®and subsequently exported as an STL file for additive manufacturing via a 3D printer. To ensure integrity, all connections were meticulously sealed with epoxy adhesive.

### Governing equations and setup of the circuit

Before diving into the methodology for deriving the output voltage versus time graph, it is imperative to grasp the foundational concept of the circuit from which the output voltage will be measured. The relationship between the current and voltage across a capacitor is represented by

2 $$\begin{aligned} {i} = C \frac{d{v}}{dt}, \end{aligned}$$

where ${i}$ represents the instantaneous current traversing the capacitor, ${C}$ denotes the capacitance *F*, and *dv*/*dt* signifies the instantaneous rate of voltage change in volts per second. As previously articulated, the principal equation governing capacitance is expressed as

3 $$\begin{aligned} {C} = \frac{\varepsilon {A}}{d} \quad \text {[F]}. \end{aligned}$$

**Fig. 5:**
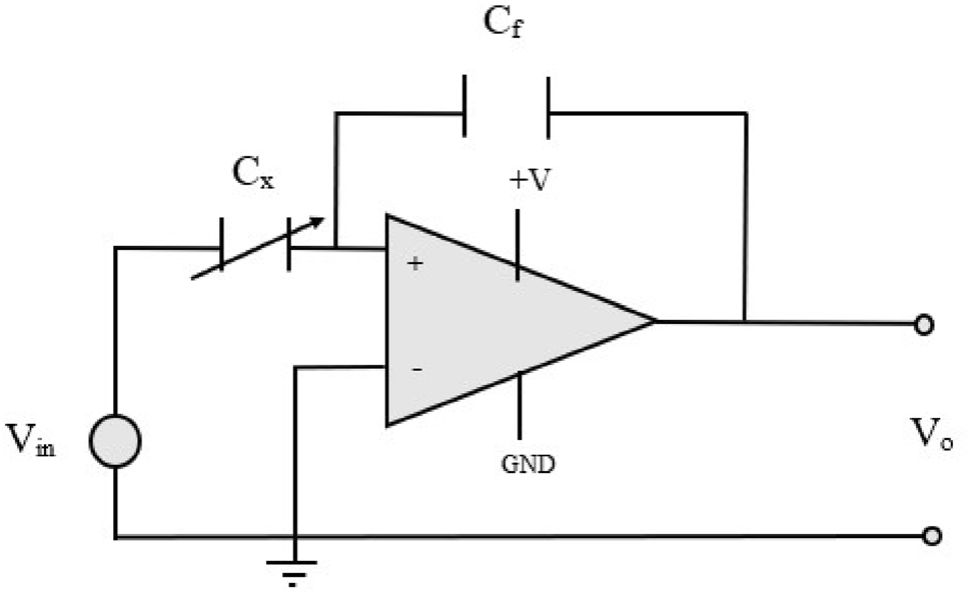
Capacitive transducer circuit.

Modifying the dielectric medium within the capacitive transducer (i.e., altering the percentage of vapor) results in a variation of the dielectric constant, $\kappa$^6^. Which in turn affects the capacitance value. This introduces a non-linear dynamic between the current and voltage. To address this, a LM348N operational amplifier (op-amp) circuit is employed as shown in Fig. 5 to linearize these relationships. Through nodal analysis of the capacitive transducer circuit, one can comprehend the principle of linearization.

Assuming an ideal op-amp, the governing equation is

4 $$\begin{aligned} \frac{{V}_0}{{V}_{{in}}} = -\frac{\varepsilon _0 \varepsilon _r {A}}{{C}_f {d}} . \end{aligned}$$

where $\varepsilon _0$ represents the absolute permittivity, $\varepsilon _r$ represents the relative permittivity or dielectric constant. $C_f$ is the capacitance, distance $d$ is the distance between the capacitive plates, $A$ is the electrode area. The justification for utilizing linearization is grounded in its ability to simplify analysis, enhance the identification of relationships between variables, and facilitate comprehension. Consequently, when data exhibits linearity, it is possible to construct an optimal fitting line and compute its slope, which serves as a measure of sensitivity.

In the experimental setup, a connection is established between a frequency generator with an amplitude of 14 V and a frequency of 50 kHz, as shown in Fig. 7. The key parameters are as shown in Table 2.

**Table 2:** Key parameters.

Parameter	Symbol	Value	Units
Capacitance	$C_f$	0.1	$\mu$F
Distance	$d$	2	cm
Electrode area	$A$	12.18	cm$^2$

These parameter values were determined through iterative experimentation until a satisfactory output waveform on the oscilloscope was achieved. It is essential to note that altering these values is possible, but caution must be exercised. Specifically:

- The amplitude voltage should always remain below or equal to the positive and negative voltage supply rails $+V_{cc}$ and $-V_{cc}$ of the op-amp. These supply rails define the upper and lower limits of the output voltage^15^.
- Adherence to the data sheet specifications^16^ of the op-amp is crucial to prevent damage or unreliable output. Parameters such as the maximum and minimum supply voltage to the rails (in this case, $\pm 15\,\textrm{V}$ for the positive rail) should be strictly followed.

Subsequently, a multimeter and an oscilloscope were connected to measure and observe the AC output voltage. The experimental arrangement, as illustrated in Fig. 7, involved placing a conical flask on an electric heater set to a constant temperature of 250 $^{\circ }$C. The recorded voltage and volume versus time are then plotted in a spreadsheet, consistent with the mathematical model.

**Fig. 6:**
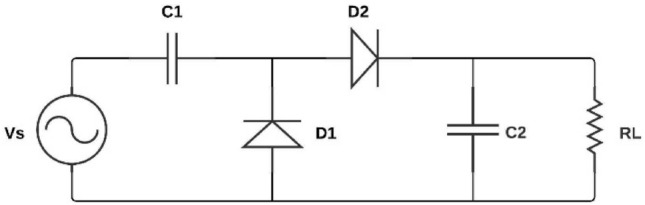
DC voltage doubler circuit.

Upon analyzing the data, it is observed that the AC output voltage from the capacitive transducer circuit exhibited low amplitude variations. To enhance the signal for ease of analysis, a multiplier circuit is introduced. This circuit amplifies the output voltage, rendering it more observable. Subsequently, it converts the amplified AC output to DC to interface it with an Arduino ®Uno® for plotting the output curve in LabVIEW ®. This combination of amplification and rectification is commonly referred to as a voltage multiplier circuit, as depicted in Fig. 6.

The experimental approach involved concise parameter selection, adherence to op-amp specifications, and subsequent signal enhancement to facilitate accurate analysis.

Upon integrating the multiplier circuit, the experimental configuration involves a signal generator operating at 14 V with a frequency of 50 kHz. This generator is connected to the capacitive transducer circuit. The subsequent steps are as follows:

- Capacitive transducer circuit: The output from this circuit serves as the input to the voltage multiplier circuit. The capacitive transducer circuit is responsible for capturing the desired signal.
- Voltage multiplier circuit: This circuit, in conjunction with a rectifier, converts the AC output from the capacitive transducer into a DC voltage. Subsequently, the voltage is amplified using the multiplier. The resulting output is a boosted voltage suitable for further analysis.
- Arduino ®Uno ®interface: The amplified output from the voltage multiplier circuit is connected to an Arduino ®Uno ®to facilitate data acquisition and processing. To protect the 5 V Arduino system from the 15 V front-end, several safety measures are implemented: a 5.1 V Zener diode clamps the analog input voltage; a 1 M$\Omega$ series resistor limits the input current; the Arduino is USB-powered separately from the 15V supply; and a high-pass filter is added before the Arduino input to suppress low-frequency noise and DC offsets, improving signal integrity and minimizing the risk of long-duration over-voltage at the ADC pin.
- LabVIEW ®visualization: The input voltage, now in DC form, is fed into LabVIEW ®. In this scenario, a voltage versus time plot is created to visualize the behavior of the system.

**Fig. 7:**
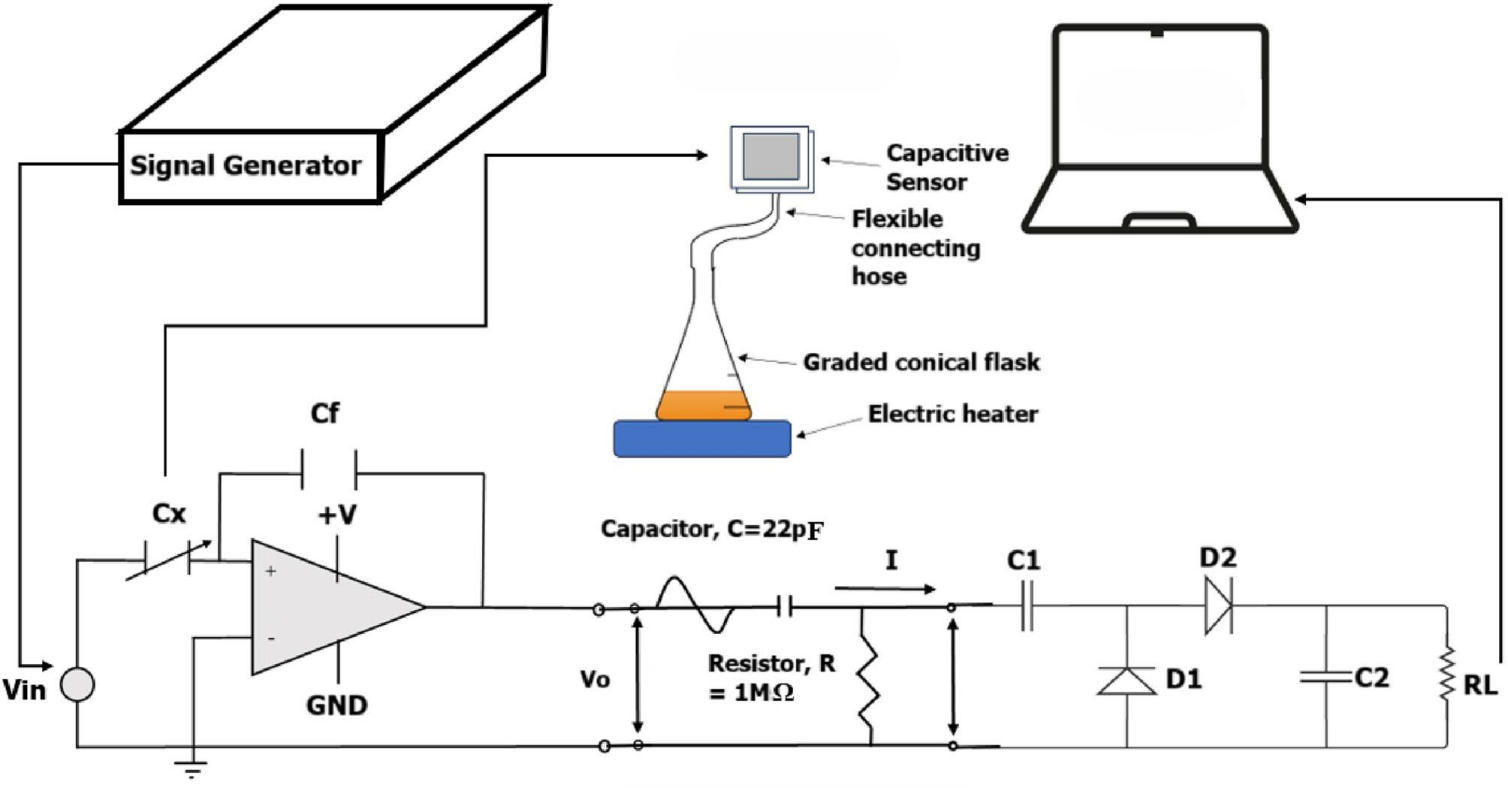
The previous setups connected all together.

Refer to Fig. 7 for a visual representation of the entire experimental setup, including the signal generation, signal processing, capacitive transducer circuit, low-pass filter circuit, and the volumetric setup.

After manual data collection, the data was inserted into a spreadsheet as seen in Table 3.

**Table 3:** Volume versus time manually measured values.

Experiment	Time [mins]	Volume [mL]
1	13, 26, 39, 52, 65	9, 7.5, 5, 4, 2
2	15, 20, 30, 40, 50	14, 12.5, 10, 9, 7
3	10, 20, 30, 40, 50	12, 10, 9, 7.5, 6
4	10, 20, 30, 40	7, 5.5, 5, 4, 1
5	20, 40, 60, 80, 100	17, 14, 7.5, 5, 3.5

## Results

The data was plotted afterwards into a best-fit line to obtain its equations.

**Fig. 8:**
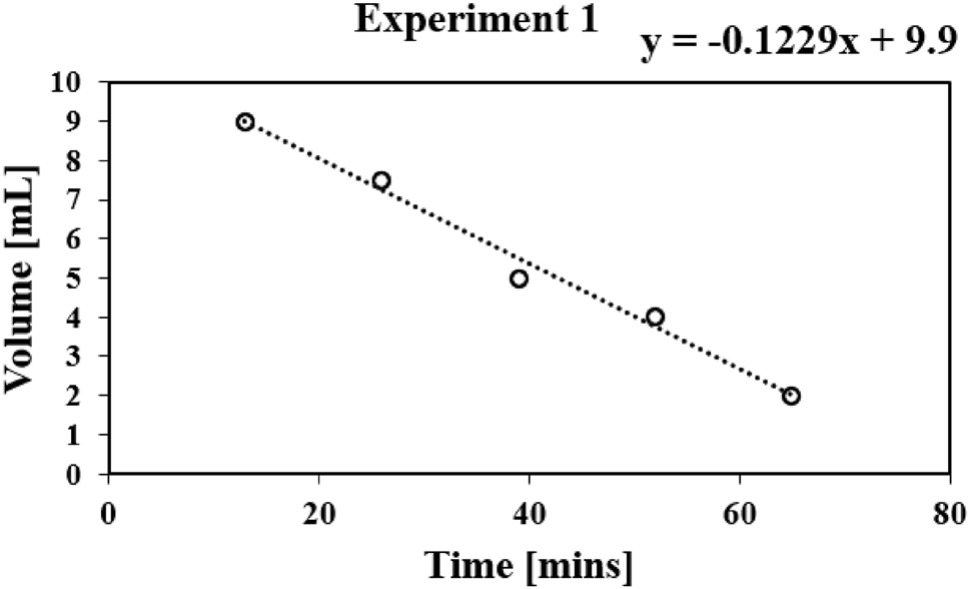
Plot demonstrating volume versus time for experiment 1.

In analyzing the 10 mL experiment within the context of the five volume versus time experiments (as depicted in Fig. 8), several key observations emerge. Notably, the initial discernible volume change occurs at the 13-minute mark, while the fifth reading is taken at the 65th minute, with a remaining volume of 2 mL. Throughout this trial, the volume change fluctuates within the range of 1 mL to 2.5 mL.

**Fig. 9:**
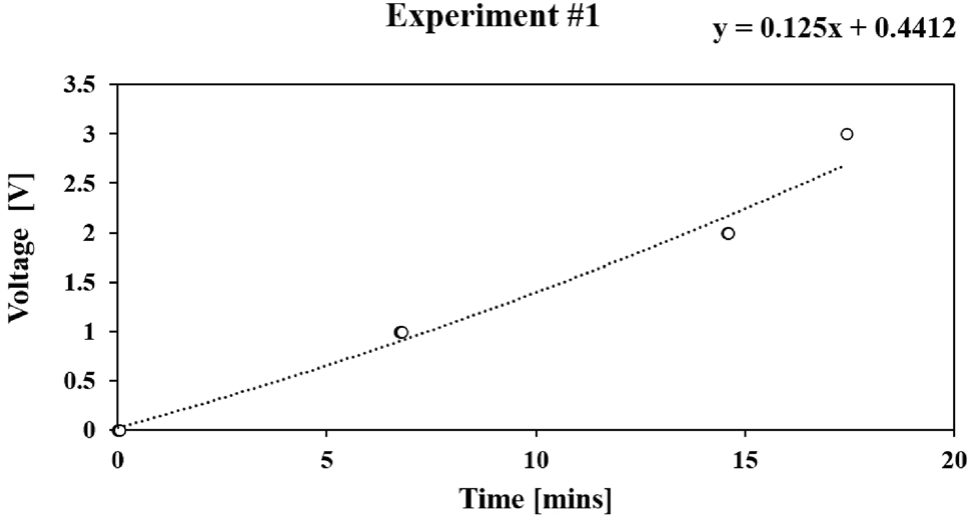
The same volume versus time for experiment 1. However, plotting voltage versus time.

Upon graphing the data and determining the best fit straight line (BFSL), a gradient of $-0.1229$ mL/min is calculated. The volume versus time plots reveal a consistent reduction in water volume throughout the experiment. These observable volumetric changes serve as essential data points for subsequent voltage versus time experiments, as seen in Fig. 9.

Within the context of the five voltage versus time experiments, this article focuses on a specific trial. The initial experiment within this set exhibits the following behavior:

- At approximately the 7-minute mark, the voltage abruptly jumps to 1 V.
- Subsequently, at the 15-minute mark, it further increases to 2 V.
- A subsequent leap occurs at the 19th minute, reaching 5 V.

Remarkably, the voltage remains stable at this level for nearly 30 minutes, indicating saturation within the capacitive transducer sensor. Upon constructing the BFSL, a gradient of *0.125* V/min is calculated. This gradient serves as a measure of sensitivity in this particular experiment.

A simulation was set up on the Falstad circuit simulator to further analyze the energy harvesting capability of the system.

**Experimental result (Real Life):**

In our prototype, a 22 pF ceramic capacitor was charged to 5 V after saturation under ambient humidity conditions. The corresponding stored energy is:

5 $$\begin{aligned} E = \frac{1}{2} \times 22 \times 10^{-12} \times (5)^2 = \frac{1}{2} \times 22 \times 10^{-12} \times 25 = 275 \times 10^{-12} = 275 \, \text {pJ} \end{aligned}$$

This reflects the very low power generation of the sensor, which we emphasize as a proof-of-concept for future development.

**Simulation result:** Using a half-wave voltage multiplier circuit with 1 mF capacitors and 50 Hz AC input, the output capacitor reached 8.662 V. The stored energy was:

6 $$\begin{aligned} E = \frac{1}{2} \times 1 \times 10^{-3} \times (8.662)^2 \approx 37.5 \, \text {mJ} \end{aligned}$$

Assuming a charging time of 2 s, this results in an approximate output power of:

7 $$\begin{aligned} P = \frac{37.5 \times 10^{-3}}{2} = 18.75 \, \text {mW} \end{aligned}$$

**Fig. 10:**
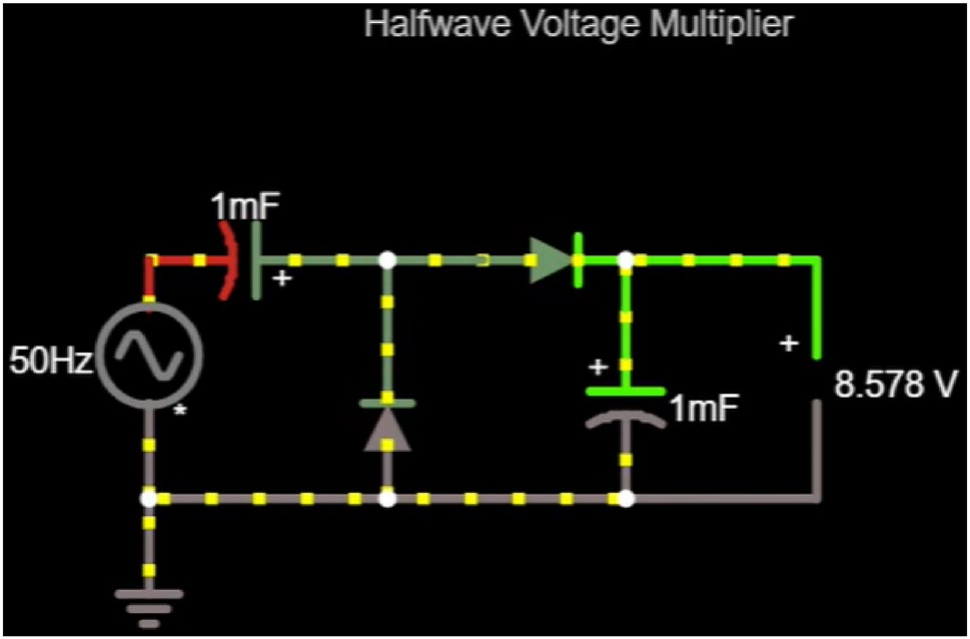
Simulation of the voltage multiplier circuit on falstad online simulation platform.

The simulation of the voltage multiplier circuit can be seen in Fig. 10.

**Fig. 11:**
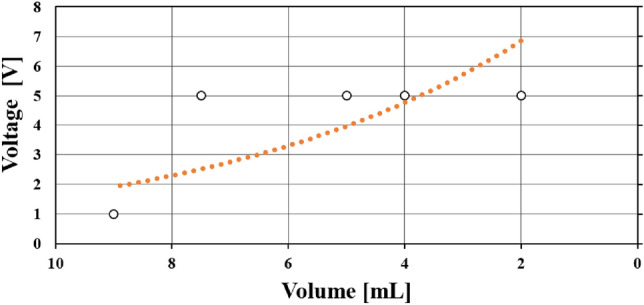
The same volume versus time for experiment 1. However, plotting voltage versus volume.

Finally, in this experiment, a correlation between the voltage and volume data was conducted by plotting voltage versus volume. The resulting curve for the 10 mL experiment within each voltage versus time plot is depicted in Fig. 11.

The measuring system starts by configuring serial communication parameters to establish reliable data transfer between the sensor and processing unit. It continuously monitors the serial buffer to prevent overflow, extracts critical analog readings and timing data, and organizes them into structured clusters for real-time graphical visualization.

The program begins by configuring the COM port for serial communication, setting parameters such as the baud rate, data mode, parity, and timeout. Once configured, the main loop reads data from the COM port, specifically 1000 bytes, but since it is set to read until a termination character, it will retrieve only the specified string format. A property node is then used to check the remaining bytes in the serial buffer to ensure it stays within bounds; excessive growth could lead to overflow and data loss due to faster transmission from the device than the reading speed on the PC. The data read from the serial buffer is passed to a ’Scan from String’ block, which interprets the string based on the defined format and returns individual values. In this case, two signed 16-bit integers representing readings from analog inputs and an unsigned 32-bit integer indicating the time interval taken by the Arduino ®loop are extracted. These variables are then combined into a cluster for display on the graph. Overall, the process ensures proper handling of serial data transmission and extraction of relevant information for visualization.

## Conclusions

Capacitive humidity sensors are essential tools in environmental and industrial monitoring, but often face challenges in sensitivity, signal processing, and reliability. This study presented a redesigned capacitive humidity sensor with a reduced plate distance (1.568 mm) and increased electrode area to enhance sensitivity. To improve signal interpretation, a linearization circuit using an operational amplifier was employed, and a voltage multiplier was integrated to amplify the output signal for easier data acquisition.

While the sensor demonstrated a measurable voltage response to humidity changes, the resulting current output remains too low for powering energy-intensive applications, such as charging mobile devices. Additionally, manual data collection and experimental variability, such as residual moisture and inconsistent vapor flow, may have affected measurement accuracy.

This work serves as a proof-of-concept, demonstrating the potential of ambient moisture-driven energy harvesting. Future efforts should focus on incorporating advanced nanomaterials to improve sensitivity, automating the measurement process for real-time monitoring, and optimizing circuit design for energy-efficient operation. These improvements could open pathways for low-power self-sustaining applications in IoT systems, environmental sensing, and climate-related studies.

### Limitations and future improvements

Several key limitations were encountered during the course of this study. Residual moisture from previous experiments could have affected the initial capacitance readings, compromising the accuracy of the measurements. While we attempted to mitigate this by exposing the sensor to sunlight between experiments to allow evaporation, this manual process introduced potential variability.

Additionally, the manual setup for introducing water vapor into the capacitive sensor made it challenging to precisely control the flow and concentration of the vapor. Fluctuations in the vapor delivery could have led to inconsistencies in the sensor response. Furthermore, the need to manually record volume and time data introduced potential human error and reduced the overall repeatability of the experiments.

To address these limitations, future work should focus on implementing a more robust drying mechanism to ensure a consistent starting point for each experiment, reducing the impact of residual moisture. Additionally, developing a controlled vapor delivery system, potentially using microfluidic components, could help precisely regulate the humidity input to the sensor. Finally, automating the data collection process, either through integrated sensors or a computer-controlled experimental setup, would enhance repeatability and reduce manual errors.

By addressing these limitations, the overall reliability and reproducibility of the sensor performance evaluation can be improved, leading to more robust conclusions and a better understanding of the system’s capabilities and constraints.

## Data Availability

The datasets used and/or analysed during the current study would be available from the corresponding author on reasonable request.
